# Solid-Phase Microextraction and the Human Fecal VOC Metabolome

**DOI:** 10.1371/journal.pone.0018471

**Published:** 2011-04-08

**Authors:** Emma Dixon, Cynthia Clubb, Sara Pittman, Larry Ammann, Zeehasham Rasheed, Nazia Kazmi, Ali Keshavarzian, Pat Gillevet, Huzefa Rangwala, Robin D. Couch

**Affiliations:** 1 Department of Chemistry and Biochemistry, George Mason University, Manassas, Virginia, United States of America; 2 Department of Computer Science and Engineering, George Mason University, Fairfax, Virginia, United States of America; 3 Department of Environmental Science and Policy, George Mason University, Manassas, Virginia, United States of America; 4 Department of Medicine, Rush University Medical Center, Chicago, Illinois, United States of America; 5 The Microbiome Analysis Center, George Mason University, Manassas, Virginia, United States of America; Howard University, United States of America

## Abstract

The diagnostic potential and health implications of volatile organic compounds (VOCs) present in human feces has begun to receive considerable attention. Headspace solid-phase microextraction (SPME) has greatly facilitated the isolation and analysis of VOCs from human feces. Pioneering human fecal VOC metabolomic investigations have utilized a single SPME fiber type for analyte extraction and analysis. However, we hypothesized that the multifarious nature of metabolites present in human feces dictates the use of several diverse SPME fiber coatings for more comprehensive metabolomic coverage. We report here an evaluation of eight different commercially available SPME fibers, in combination with both GC-MS and GC-FID, and identify the 50/30 µm CAR-DVB-PDMS, 85 µm CAR-PDMS, 65 µm DVB-PDMS, 7 µm PDMS, and 60 µm PEG SPME fibers as a minimal set of fibers appropriate for human fecal VOC metabolomics, collectively isolating approximately 90% of the total metabolites obtained when using all eight fibers. We also evaluate the effect of extraction duration on metabolite isolation and illustrate that *ex vivo* enteric microbial fermentation has no effect on metabolite composition during prolonged extractions if the SPME is performed as described herein.

## Introduction

Volatile organic compounds (VOCs) are a large and highly diverse group of carbon-based molecules, generally related by their volatility at ambient temperature. The diagnostic potential of VOCs in biological specimens has begun to receive considerable attention and correlations between the VOC metabolome and various diseases are emerging [1]–[9]. Specialized headspace sampling methods, such as solid-phase microextraction (SPME), have greatly facilitated the isolation of VOCs from biological specimens [10]–[13]. A typical headspace SPME analysis involves the extraction of the VOCs via partitioning into a polymeric coating adhered to a fused silica rod (fiber), subsequent desorption of the VOCs by heating the fiber in the injection port of a gas chromatograph, separation of the VOCs by gas-liquid partition chromatography, and detection of the VOCs via flame ionization and/or mass spectrometry.

The duration of extraction and choice of polymeric coating are two important considerations when performing a SPME analysis. Ideally, a quantitative analysis is performed when the analyte distribution is in equilibrium between the sample and the SPME fiber coating, such that small deviations in extraction duration do not significantly alter analyte titers [14]. Several SPME fiber coatings are currently commercially available, including polyacrylate (PA), polydimethylsiloxane (PDMS), carbowax-polyethylene glycol (PEG), and mixed phases of carboxen (CAR)-PDMS, divinylbenzene (DVB)-PDMS, and CAR-DVB-PDMS. While the polarity of the analyte of interest is typically used to guide the selection of a particular SPME fiber [13], metabolomic analyses generally strive to isolate and identify numerous, chemically diverse types of analyte molecules. Thus, a single SPME fiber coating may be insufficient for a comprehensive analysis of the complex analyte mixtures typically present in biological samples. The multifarious nature of biological sample composition also complicates the SPME procedure, as compounds with higher affinity for the fiber may compete with and displace those with lower affinity [15].

Abnormally foul smelling stools often accompany gastrointestinal disease. Using headspace SPME with a CAR-PDMS fiber, pioneering investigations have begun to explore the diagnostic and health implications of VOCs isolated from human feces [3], particularly as they relate to pathogenic disease [2], [4], [5]. However, we hypothesized that the diverse nature of metabolites present in human feces dictates the use of several different SPME fiber coatings for more comprehensive metabolomic coverage. Thus, to further the development of human fecal VOC metabolomics, we describe here our assessment of eight different commercially available SPME fibers for their ability to isolate and quantitate VOCs from human feces. In combination with both GC-MS and GC-FID, we illustrate the effects of extraction duration and SPME fiber type on the number and type of metabolites isolated. Overall we illustrate that a comprehensive analysis requires the use of all eight SPME fiber types. However, approximately 90% coverage is achieved using five of the fibers, better accommodating sample throughput.

## Materials and Methods

### Fecal samples

Fecal samples (∼25 g) were collected from asymptomatic donors and, within 1 hour of passage, sealed in a 50 mL sterile conical tube then frozen by immersing the tube in liquid nitrogen. The frozen samples were dispensed in 0.2 g aliquots into 4 mL WISP style screw thread amber glass vials and sealed with Black Top Hat PTFE/Silicone caps (J.G. Finneran, Vineland, NJ). All samples were stored at −80°C until analyzed. This study was approved by the Institutional Review Boards at George Mason University and Rush University Medical Center. Study participants provided written informed consent.

### Headspace solid-phase microextraction procedure

Sample vials were heated to 60°C for 30 minutes then a SPME fiber assembly was manually positioned into the headspace above the feces and the fiber exposed to the volatiles for the duration indicated (the sample vial temperature was held at 60°C for the duration of the exposure). Extraction conditions were tightly controlled to ensure reproducible and quantitative SPME results, particularly with nonequilibrium extraction durations [16]. The fiber assembly was then placed into the GC inlet for thermal desorption of the analytes. The following SPME fibers (Supelco, Bellefonte, PA) were used in our investigation: DVB-PDMS 65 µm, PA 85 µm, CAR-PDMS 75 µm, CAR-PDMS 85 µm, PDMS 100 µm, PDMS 7 µm, PEG 60 µm, and CAR-DVB-PDMS 50/30 µm. All fibers were preconditioned before use, as per the manufacturer's instructions. After every sample analysis, the fiber was reanalyzed (without exposure to a sample) to ensure complete desorption of analytes. All analyses were performed in duplicate.

### Instruments

Samples were analyzed using an Agilent 7890A GC and 5975C inert XL mass selective detector (MSD) with triple axis detector (Agilent, Palo Alto, CA) or an Agilent 6890 Plus GC-FID. The GC-MS was equipped with a DB5-MS capillary column (Agilent), 30 m in length, 0.25 mm ID, and 0.25 µm film thickness, and a 0.75 mm ID SPME injection port liner operated in splitless mode at varying inlet temperatures, dependent upon the SPME fiber used (see Table 1). The GC-FID was equipped with a DB5-MS capillary column (Agilent, Palo Alto, CA), 15 m in length, 0.25 mm ID, and 0.25 µm film thickness, and a 0.75 mm ID SPME injection port liner operated in splitless mode at varying inlet temperatures (Table 1).

**Table 1:** GC Inlet temperatures per fiber type.

SPME Fiber	GC-MS and GC-FID Inlet Temperature (°C)
65 µm DVB-PDMS	250
85 µm PA	280
75 µm CAR-PDMS	300
85 µm CAR-PDMS	280
100 µm PDMS	250
7 µm PDMS	320
60 µm PEG	240
50/30 µm CAR-DVB-PDMS	270

### GC-MS and GC-FID conditions

For the GC-MS, helium carrier gas was set to 1.17 mL/min flow rate and the GC oven was held at an initial temperature of 35°C for 1 min, ramped to 80°C at 3°C/min, then to 120°C at 10°C/min, and finally to 260°C at 40°C/min. The final temperature of 260°C was held for 1.5 min. The total run time for the analysis method was 25.0 min. The Agilent 5975C MSD was scanned from 30 to 550 amu at a rate of 2.81 scans/s. Compounds were identified using the National Institute of Standards and Technology (NIST, Washington, DC) Automated Mass Spectral Deconvolution and Identification System (AMDIS, ver 2.69) software and mass spectral library (NIST08).

The GC-FID system used helium carrier gas at a flow rate of 1.5 mL/min and the GC oven was held at an initial temperature of 35°C for 1 min, ramped to 80°C at 3°C/min, then to 120°C at 10°C/min, and finally to 260°C at 40°C/min. The final temperature of 260°C was held for 1.5 min. The total run time for the analysis was 25.0 min.

### Data processing

Y_max_ was defined by nonlinear regression fitting the extraction duration plot to Y = Y_max_*X/(K+X).

Heat maps were prepared from AMDIS analyzed chromatograms as follows. Only compounds with a spectral match score greater than or equal to 90% were considered. For each fiber type, the analysis was performed in duplicate and the replicate metabolite lists were combined by averaging appropriate peak areas to eliminate redundancies. A color plot matrix was generated, with the fibers as the columns and compound identity as the rows. Peak area values were related to color intensities using indole as the reference compound, as it was ubiquitous to all fibers.

Binary plots were generated using retention times from GC-MS and GC-FID chromatograms. Chromatograms were manually aligned and then peaks were partitioned into bins according to retention time values. Replicate samples were combined to eliminate redundancies. A binary matrix was generated, with the fibers as the columns and median bin value as the rows. To condense the overall size of the matrix, rows were excluded if all columns were empty. Black shading identifies the presence of a peak within a bin.

### Culturing conditions

Anaerobic and aerobic growth was evaluated by aseptically dispensing 3 mL of Luria-Bertani (LB), tryptic soy broth (TSB) supplemented with 0.1% cysteine, or Chamberlain's media into 13×100 mm glass culture tubes then inoculating using a sterile loop. The inoculum was obtained from a frozen fecal sample equilibrated to room temperature for 5 min or from frozen fecal samples pre-sterilized before use by immersion in a boiling water bath for 5 min or autoclaving for 15 min, 15 psi, 121°C (negative controls). Aerobic incubations were performed at 37°C, 250 rpm for 18 hr. Anaerobic incubations were performed by placing the culture tube into an activated GasPak EZ pouch (BD Diagnostic Systems, Sparks, MD, USA) then incubating at 37°C for 18 hr. Optical density of the cultures (OD_600_) was determined using an Agilent 8453 UV-Vis spectrophotometer. All reported values are relative to uninoculated media and are the average of duplicate experiments.

To determine colony forming units (cfu), fecal samples were heated to 60°C for the indicated duration, resuspended to an OD_600_ of 1.2 using sterile phosphate buffered saline (PBS), diluted by a series of 10-fold serial dilutions (in PBS), then 100 µL of each dilution was used to inoculate duplicate TSB+0.1% cysteine agar plates. Microbial colonies were titered after overnight incubation at 37°C.

## Results and Discussion

### Extraction duration

**Figure 1:**
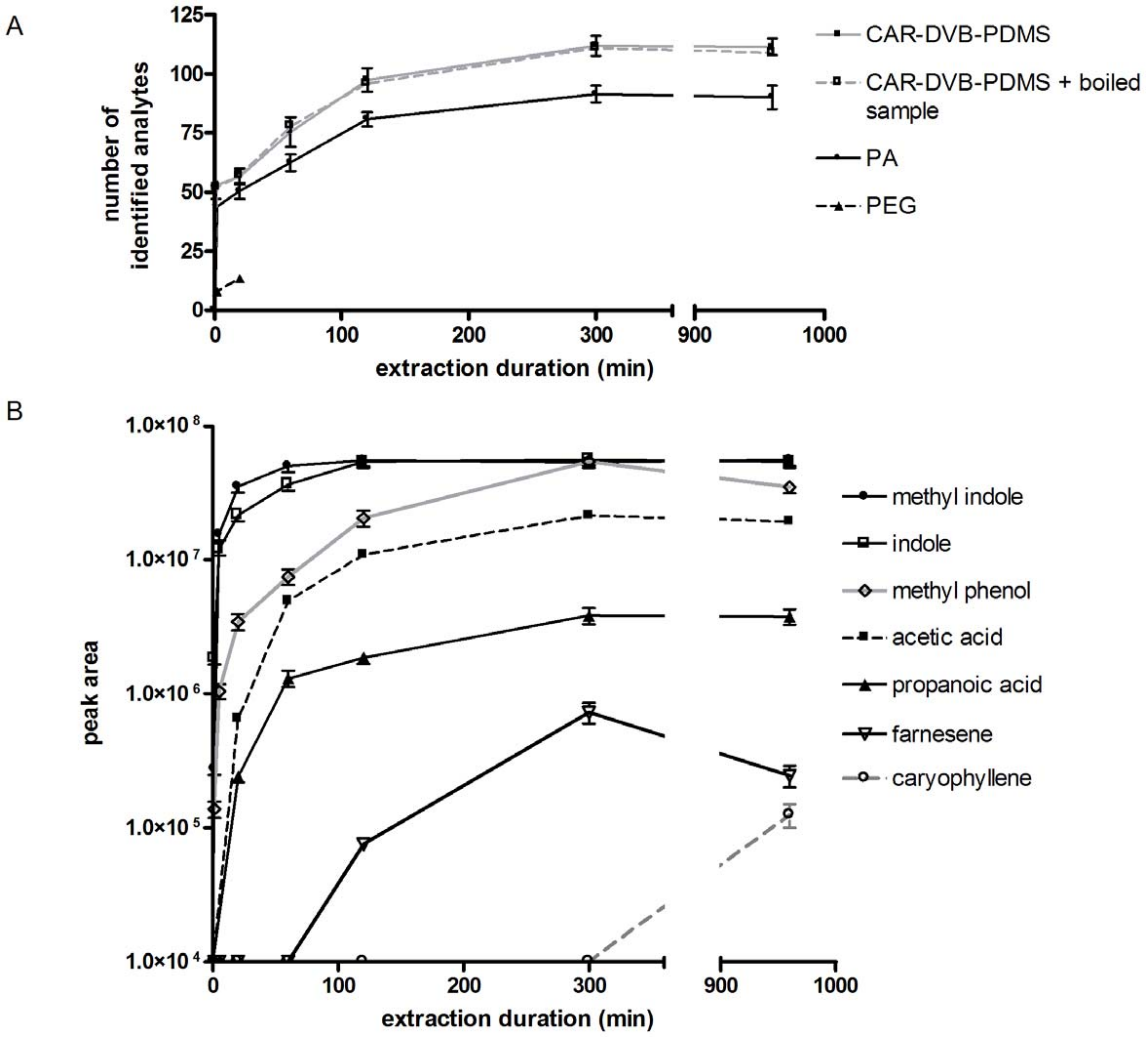
Extraction duration and headspace SPME of human feces. A) A plot of identified analytes as a function of extraction time. Nonlinear regression fitting the hyperbolic extraction curve yields a Y_max_ of 114+/−3 for the CAR-DVB-PDMS fiber (R^2^ = 0.9937) and 94+/−4 for the PA fiber (R^2^ = 0.9791). The PEG plot could not be extended beyond 20 min due to an unknown analyte overwhelming the MS detector. Also shown in this plot is the number of identified analytes obtained from a sample that was pretreated by boiling for 5 min before extraction. Essentially no difference in analyte number or composition is observed with the pretreated sample (CAR-DVB-PDMS+boiled sample) relative to an untreated sample (CAR-DVB-PDMS). All samples were analyzed in duplicate. See text for further discussion. B) A plot of area under the chromatographic curve as a function of time for the indicated analytes obtained using the CAR-DVB-PDMS fiber. Differences in extraction rates for the indicated metabolites are apparent.

To ascertain the effect of extraction duration on metabolite isolation from the headspace above human feces, we used three different SPME fibers (a 50/30 µm CAR-DVB-PDMS, 85 µm PA, and a 60 µm PEG fiber) in conjunction with GC-MS to identify and quantify the volatile metabolites. To perform the analysis, samples were heated to 60°C for 30 min, then a SPME fiber was placed into the headspace above the sample for various time intervals (ranging from 1 min to 16 hr; the temperature was held constant at 60°C throughout the extraction). Extracted analytes were then immediately desorbed into a GC-MS system and spectral comparison with the NIST08 database facilitated analyte identification. Only compounds with a 90% or greater probability of match to a molecule in the NIST08 library were scored. Figure 1a illustrates a plot of the number of analytes identified as a function of extraction duration. As observed in the Figure, regardless of the extraction duration, the CAR-DVB-PDMS fiber isolated a greater number of identifiable analytes from the sample than did the other tested fibers, highlighting the influence of fiber choice on fecal VOC metabolomics (further addressed below). For both the PA and CAR-DVB-PDMS fibers, total analyte extraction appears hyperbolic, with a near maximum value (Y_max_) occurring with a 960 minute extraction duration (98% of Y_max_). In contrast, prolonged extractions with the PEG fiber are problematic, as an unidentified metabolite floods and overwhelms the detector at extraction durations beyond 20 min. As illustrated with the CAR-DVB-PDMS fiber (Fig. 1b), individual analyte extraction rates are analyte specific, with some metabolites (such as indole and methyl indole) rapidly reaching equilibrium and others (such as acetic acid, propanoic acid, and caryophyllene) proceeding more slowly. In some cases (such as observed with methyl phenol and farnesene), metabolite titers plateau then subsequently wane with increased exposure duration, a phenomena attributed to higher affinity compounds displacing those with lower affinity for the fiber, thereby lowering the titer of the latter [15].

**Figure 2:**
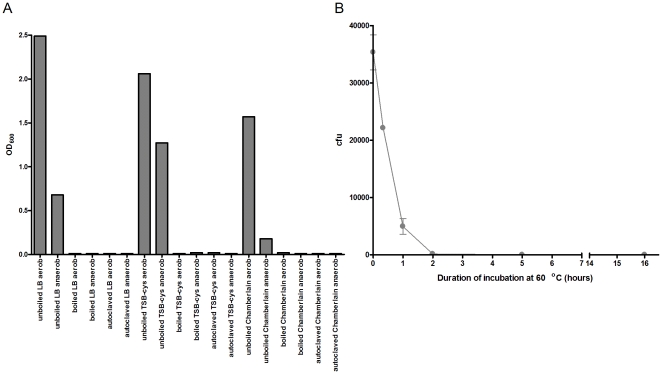
Heat sterilization of human feces. A) Human fecal aliquots, dispensed in vials, were either autoclaved or placed in a boiling water bath then used as inoculum for liquid cultures incubated aerobically or anaerobically, as described in Materials and Methods. While untreated (not autoclaved or boiled) fecal samples display growth in all three media compositions (LB – Luria Bertani media, TSB-cys – tryptic soy broth +0.1% cysteine media, Chamberlain – Chamberlain media) and culturing conditions, autoclaving or boiling the samples abolishes growth. Reported values are average of duplicates. B) Aliquots of a human fecal sample were incubated at 60°C for the indicated duration then used as inoculum for LB-agar plates, as detailed in Materials and Methods. A plot of colony forming units (cfu) as a function of incubation duration illustrates the loss of enteric microbial viability over the first hour of 60°C incubation, with no growth observed after 2 hours and beyond. The experiment was performed in duplicate.

With the exception of the PEG fiber, Fig. 1 illustrates how the number of identified analytes increases with extended extraction duration, reflecting changes in chromatographic peak areas as well as the appearance of additional peaks in the chromatograms obtained with prolonged extractions. To determine if enteric microbial fermentation occurs during these prolonged extractions, potentially contributing towards the total number of identified analytes (produced *ex vivo*), we compared the VOCs isolated from unboiled and boiled (sterile) fecal samples and also evaluated the survival of enteric microbes at 60°C. As shown in Fig. 2a, liquid culture media inoculated with boiled or autoclaved fecal samples are devoid of growth under both anaerobic and aerobic conditions, in contrast to cultures inoculated with unboiled samples. Subsequent SPME analysis of the boiled and unboiled samples (60°C extraction; 50/30 µm CAR-DVB-PDMS fiber) identifies no discernible difference in either the total number of headspace analytes or the analyte composition (Fig. 1a). Additionally, as shown in Fig. 2b, when the fecal sample is heated to 60°C, the number of viable enteric microbes rapidly declines (90% of microbial viability is lost after 1 hr), with near complete absence of viability observed after 2 hours of heating. Collectively, the results indicate that when headspace SPME of human feces is performed at 60°C, 99.5% of enteric microbial viability is lost after 2 hrs and *ex vivo* microbial fermentation does not significantly contribute to the headspace VOC composition. Therefore, a 16 hr extraction duration (fiber type permitting) appears well suited to a metabolomic analysis aimed at isolating and identifying the maximum number of analytes. An analyte-specific quantitative analysis, on the other hand, may require individual optimization, as competitive displacement may significantly affect metabolite titers.

### SPME fiber comparison

#### 1. Using GC-MS and Metabolite Identities

To permit a comparison of all eight commercially available SPME fibers, fecal samples were heated to 60°C for 30 min then the headspace was extracted with an individual fiber for 20 min (the temperature was maintained at 60°C throughout the extraction). Extracted analytes were identified by GC-MS and comparison with the NIST08 mass spectral library. Again, only those compounds with 90% or greater probability of match to a molecule in the mass spectral library were named.

**Figure 3:**
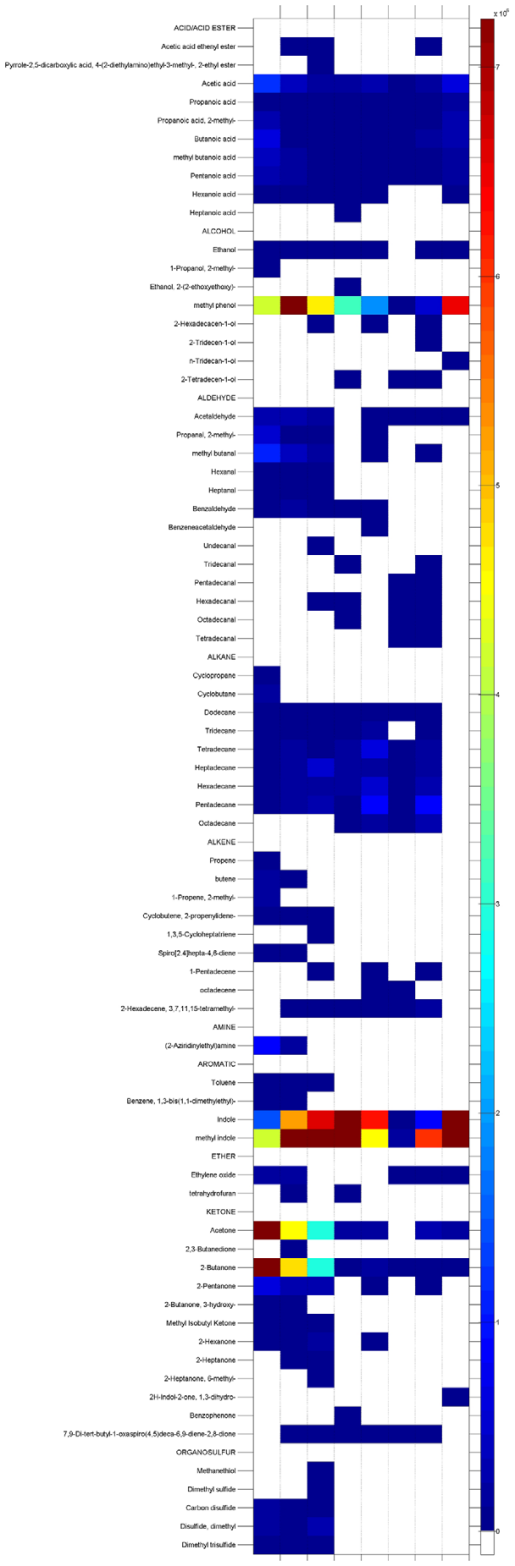
Fiber-dependent GC-MS identification and analysis of metabolites present in the human fecal VOC metabolome. A heat map presents the identified metabolites extracted by each SPME fiber and their relative chromatographic peak areas. Each of the eight columns in the heat map represents a different fiber. Metabolites are indicated on each row and are organized by functional group. Each extraction was performed in duplicate and replicates combined by averaging peak area values. Fiber legend: A - 75 µm CAR-PDMS, B - 85 µm CAR-PDMS, C - 50/30 µm CAR-DVB-PDMS, D - 85 µm PA, E - 65 µm DVB-PDMS, F - 7 µm PDMS, G - 100 µm PDMS, H - 60 µm PEG.

**Figure 4:**
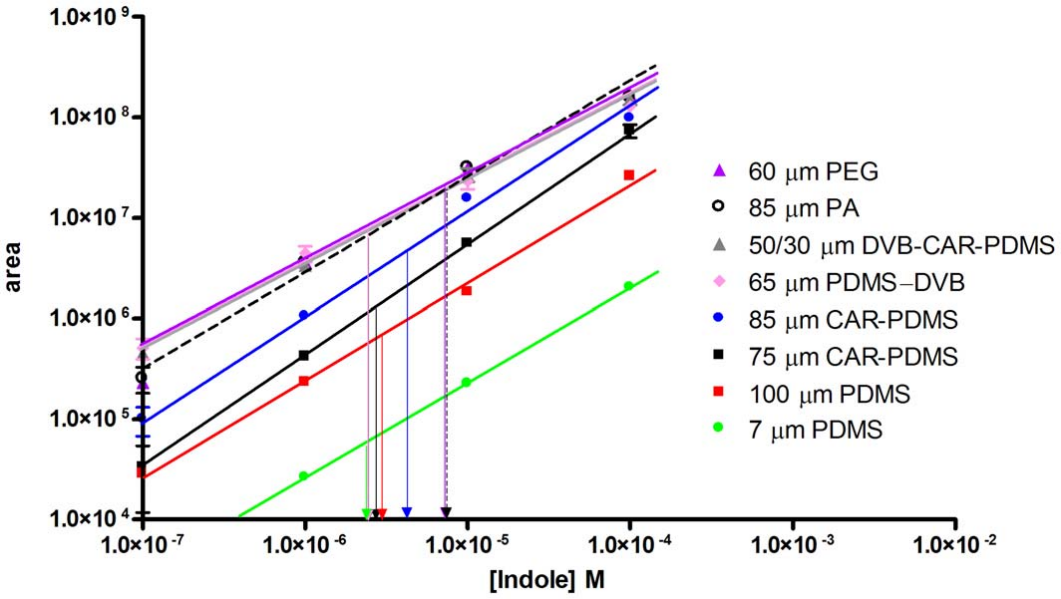
Indole standard curves generated by headspace SPME. Chromatographic peak area is plotted as a function of indole concentration, illustrating fiber-dependent differences in the partition coefficient for indole. Peak areas taken from Fig. 3 were used to determine the concentration of indole extracted using each fiber (arrows).

As illustrated in Fig. 3, under the extraction conditions employed, a total of 73 different compounds were identified. Overall, the 73 compounds encompass a wide range of molecular weight and polarity and belong to 10 chemical classes: alcohols (8), ketones (12), aldehydes (13), acid/acid esters (10), amines (1), ethers (2), organosulfurs (5), aromatics (4), alkanes (9), and alkenes (9). Compounds such as acetic acid, propanoic acid, butanoic acid, pentanoic acid, methyl phenol, indole, methyl indole, and butanone are ubiquitously isolated from the sample, regardless of the fiber type. For many of these, fiber-dependent differences in partition coefficients are considerable. Indole, for example, displays a 200 fold difference in peak area among the fiber types (Fig. 3). Indole standard curves indicate that a near identical amount of indole (2.4–7.4 µM) was extracted from the sample using each of the tested fibers (Fig. 4), underscoring the importance of standard curves when performing a quantitative, multi-fiber comparative analysis.

In terms of total number of metabolites identified, fiber-dependent differences are also observed. Of the 73 total compounds, the 50/30 µm CAR-DVB-PDMS fiber affords the greatest proportion, with 47 associated molecules, 6 of which are uniquely obtained with that fiber (Table 2). The 85 µm and 75 µm CAR-PDMS fibers perform similarly, isolating 46 and 45 total identified metabolites, respectively. Collectively, the results suggest that a human fecal metabolomic analysis intent on obtaining the maximum number of different VOCs requires the use of all but one of the SPME fibers tested (the 7 µm PDMS fiber), as this lone fiber does not appear to contribute any unique metabolites to the collective. Alternatively, combinatorial analysis and comparison of optimal fiber combinations identifies a three-, four-, and five-fiber grouping that affords substantial (89–96%) coverage of the total metabolites identified (Table 3) while reducing the number of extractions required, thereby enhancing the throughput of sample analysis. However, before defining an ideal fiber combination, the presence of numerous additional, unidentified peaks associated with each fiber prompted us to compare these results to those obtained from an analysis using peak retention times.

**Table 2:** Fiber-dependent extraction of metabolites.

SPME Fiber	GC-MS (Identities)		GC-MS (Retention Time)		GC-FID (Retention Time)	
	total^a^	unique^b^	total	unique	total	unique
75 µm CAR-PDMS	45 (60)	5	93 (35)	10	109 (50)	6
85 µm CAR-PDMS	46 (62)	1	119 (44)	15	119 (54)	6
50/30 µm CAR-DVB-PDMS	47 (63)	6	107 (41)	13	157 (71)	16
85 µm PA	32 (44)	3	53 (20)	8	82 (37)	4
65 µm DVB-PDMS	32 (45)	1	122 (46)	21	138 (63)	8
7 µm PDMS	28 (36)	0	90 (34)	20	76 (35)	5
100 µm PDMS	29 (49)	1	98 (37)	9	80 (37)	1
60 µm PEG	18 (26)	2	74 (28)	12	88 (41)	8

a bracketed values are the percentage of total metabolites identified.

b number of metabolites exclusively associated with the fiber type.

**Table 3:** Top fiber combinations.

Optimal SPME Fiber or Fiber Combination^a^	GC-MS(Identities)^b^	GC-MS(Retention Time)	GC-FID(Retention Time)
single fiber	50/30 µm CAR-DVB-PDMS (63)	65 µm DVB-PDMS (46)	50/30 µm CAR-DVB-PDMS (71)
two fiber combination	50/30 µm CAR-DVB-PDMS75 µm CAR-PDMS (78)	65 µm DVB-PDMS85 µm CAR-PDMS (63)	50/30 µm CAR-DVB-PDMS85 µm CAR-PDMS (80)
three fiber combination	50/30 µm CAR-DVB-PDMS75 µm CAR-PDMS85 µm PA (89)	65 µm DVB-PDMS85 µm CAR-PDMS7 µm PDMS (75)	50/30 µm CAR-DVB-PDMS85 µm CAR-PDMS65 µm DVB-PDMS (86)
four fiber combination	50/30 µm CAR-DVB-PDMS75 µm CAR-PDMS85 µm PA100 µm PDMS (93)	65 µm DVB-PDMS85 µm CAR-PDMS7 µm PDMS60 µm PEG (82)	50/30 µm CAR-DVB-PDMS85 µm CAR-PDMS65 µm DVB-PDMS7 µm PDMS (91)
five fiber combination	50/30 µm CAR-DVB-PDMS75 µm CAR-PDMS85 µm PA100 µm PDMS65 µm DVB-PDMS (96)	65 µm DVB-PDMS85 µm CAR-PDMS7 µm PDMS60 µm PEG50/30 µm CAR-DVB-PDMS (88)	50/30 µm CAR-DVB-PDMS85 µm CAR-PDMS65 µm DVB-PDMS7 µm PDMS60 µm PEG (94)
six fiber combination	50/30 µm CAR-DVB-PDMS75 µm CAR-PDMS85 µm PA100 µm PDMS65 µm DVB-PDMS60 µm PEG (99)	65 µm DVB-PDMS85 µm CAR-PDMS7 µm PDMS60 µm PEG50/30 µm CAR-DVB-PDMS75 µm CAR-PDMS (93)	50/30 µm CAR-DVB-PDMS85 µm CAR-PDMS65 µm DVB-PDMS7 µm PDMS60 µm PEG75 µm CAR-PDMS (97)
seven fiber combination	50/30 µm CAR-DVB-PDMS75 µm CAR-PDMS85 µm PA100 µm PDMS65 µm DVB-PDMS60 µm PEG85 µm CAR-PDMS (100)	65 µm DVB-PDMS85 µm CAR-PDMS7 µm PDMS60 µm PEG50/30 µm CAR-DVB-PDMS75 µm CAR-PDMS100 µm PDMS (97)	50/30 µm CAR-DVB-PDMS85 µm CAR-PDMS65 µm DVB-PDMS7 µm PDMS60 µm PEG75 µm CAR-PDMS85 µm PA (99)
eight fiber combination	all tested fibers (100)	all tested fibers (100)	all tested fibers (100)

a based on maximal coverage of the total metabolites identified.

b bracketed values are the percentage of total metabolites obtained using all eight fibers.

#### 2. Using GC-MS Peak Retention Times

**Figure 5:**
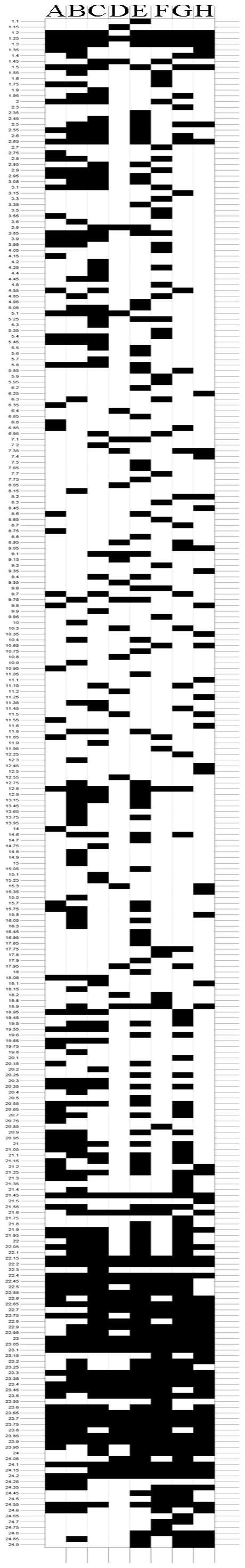
Binary plot illustrating the GC-MS chromatographic peaks (metabolites) associated with each SPME fiber. Columns represent the fibers while rows indicate peak retention times. Each extraction was performed in duplicate and replicates combined. Fiber legend: A - 75 µm CAR-PDMS, B - 85 µm CAR-PDMS, C - 50/30 µm CAR-DVB-PDMS, D - 85 µm PA, E - 65 µm DVB-PDMS, F - 7 µm PDMS, G - 100 µm PDMS, H - 60 µm PEG.

A consequence of focusing solely on identifiable compounds within the GC-MS chromatogram is that a considerable number of metabolites are overlooked. For instance, with the probability-of-match threshold set to 90%, the mass spectral deconvolution and metabolite identification software tentatively identifies an average of 41% of the total number of chromatographic peaks collectively observed in the multi-fiber analysis described above. Since differential metabolomics can be performed in the absence of peak identities, we elected to also compare the eight SPME fibers by evaluation of their total number of associated chromatographic peaks. As illustrated in Fig. 5, under the extraction conditions described above, a total of 267 chromatographic peaks are identified. The majority of these peaks are associated with the 65 µm DVB-PDMS, 85 µm CAR-PDMS, and 50/30 µm CAR-DVB-PDMS fibers, extracting 122, 118, and 108 metabolites, respectively (Table 2). In contrast to the identity-based comparison described above, all of the fiber types are associated with unique peaks, indicating that a comprehensive metabolomic analysis is achieved only when all eight of the tested fibers are used (rather than seven, as suggested above). Combinatorial analysis identifies an optimal five-fiber combination that is sufficient to achieve 88% coverage of the total chromatographic peaks (Table 3), specifically highlighting the grouping of the 50/30 µm CAR-DVB-PDMS, 85 µm CAR-PDMS, 65 µm DVB-PDMS, 7 µm PDMS, and 60 µm PEG fibers. Interestingly, these fibers represent five of the six different fiber coatings evaluated in our study. To see if this propensity is also observed with an alternative detector type, we next compared fiber-associated chromatographic peaks obtained using GC-FID.

#### 3. SPME fiber comparison using GC-FID

**Figure 6:**
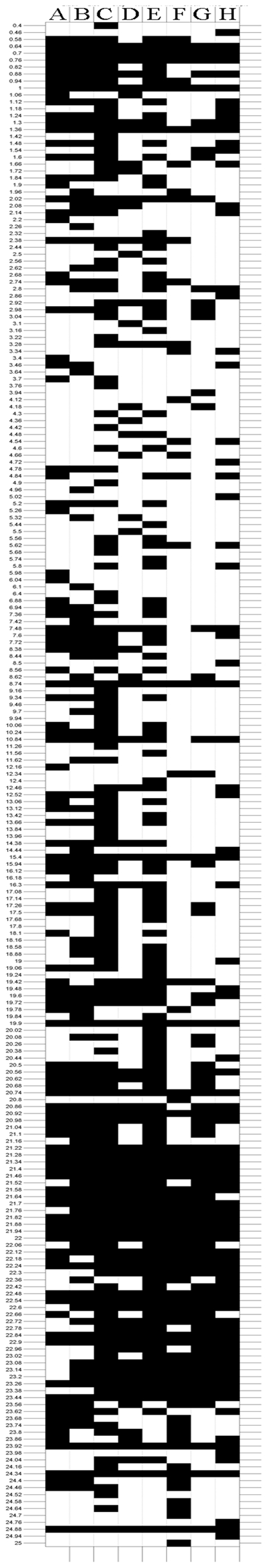
Binary plot illustrating the GC-FID chromatographic peaks (metabolites) associated with each SPME fiber. Columns represent the fibers while rows indicate peak retention times. Each extraction was performed in duplicate and replicates combined. Fiber legend: A - 75 µm CAR-PDMS, B - 85 µm CAR-PDMS, C - 50/30 µm CAR-DVB-PDMS, D - 85 µm PA, E - 65 µm DVB-PDMS, F - 7 µm PDMS, G - 100 µm PDMS, H - 60 µm PEG.

As illustrated in Fig. 6, using extraction conditions identical to those described above, a total of 267 chromatographic peaks are obtained using the eight commercially available SPME fibers in combination with GC-FID. As was observed using GC-MS, the majority of the total peaks are associated with the 50/30 µm CAR-DVB-PDMS, 65 µm DVB-PDMS, and 85 µm CAR-PDMS fibers, extracting 71%, 63%, and 54% of the total metabolites, respectively (Table 2). Every fiber tested has at least one associated unique peak, again indicating that a comprehensive analysis requires the use of all eight fibers. However, also in agreement with the GC-MS outcome, combinatorial analysis highlights the grouping of the 50/30 µm CAR-DVB-PDMS, 85 µm CAR-PDMS, 65 µm DVB-PDMS, 7 µm PDMS, and 60 µm PEG fibers to achieve significant coverage of the total chromatographic peaks (94%; Table 3) while economizing sample processing.

### Conclusions

While pioneering fecal VOC metabolomic investigations employed only a 75 µm CAR-PDMS fiber, we hypothesized that a single fiber is insufficient for a comprehensive metabolomic analysis due to the chemical diversity of analyte molecules anticipated to be present in human feces. As detailed in Tables 2 and 3, in support of our hypothesis, both GC-MS and GC-FID analyses indicate that maximum metabolite coverage requires the use of multiple fibers. While comparison of metabolite identities suggested the 7 µm PDMS fiber may be expendable, the corresponding GC-MS chromatogram contains 20 unique unidentified peaks (Table 2). Additionally, GC-FID analysis identifies at least one unique peak associated with every tested fiber, further indicating that maximal metabolite coverage requires the use of all eight commercially available fibers.

Utilizing a 16 hr extraction duration, a reasonably well equipped lab can manually process 8–10 fiber extractions per day. Thus, the number of available samples and study duration will dictate if the use of a complete fiber set is feasible. In an effort to achieve a balance between a comprehensive analysis and enhanced sample throughput, we used a combinatorial examination of the GC-MS and GC-FID results to propose fiber combinations that yield the greatest metabolite coverage (Table 4). For a GC-FID analysis, we suggest a five fiber combination that includes the 50/30 µm CAR-DVB-PDMS, 85 µm CAR-PDMS, 65 µm DVB-PDMS, 7 µm PDMS, and 60 µm PEG fibers. Collectively, these five fibers encompass 94% of the GC-FID chromatographic peaks obtained using all eight fibers (Table 4). While these same five fibers cover 88% of the total metabolites detected by GC-MS, this analysis may benefit from the addition of a 75 µm CAR-PDMS fiber, as the combination of these six fibers increases the coverage to 93%. It is noteworthy that these same fiber combinations produced similar total metabolite coverage (+/−2%) in samples obtained from three different healthy donors. Additionally, although the five fiber set comprises five of the six different fiber coatings evaluated, it is also noteworthy that the inclusion of the sixth fiber coating (85 µm PA) is not as effective as inclusion of the 75 µm CAR-PDMS fiber (Table 4).

**Table 4:** Recommended fiber combinations.

Suggested SPME Fiber Combination	Percent coverage^a^		
	GC-MS(Identities)	GC-MS(Retention Time)	GC-FID(Retention Time)
50/30 µm CAR-DVB-PDMS85 µm CAR-PDMS65 µm DVB-PDMS7 µm PDMS60 µm PEG75 µm CAR-PDMS85 µm PA	99	97	99
50/30 µm CAR-DVB-PDMS85 µm CAR-PDMS65 µm DVB-PDMS7 µm PDMS60 µm PEG75 µm CAR-PDMS^b^	93	93	97
50/30 µm CAR-DVB-PDMS85 µm CAR-PDMS65 µm DVB-PDMS7 µm PDMS60 µm PEG	87	88	94
50/30 µm CAR-DVB-PDMS85 µm CAR-PDMS65 µm DVB-PDMS7 µm PDMS	84	81	91
50/30 µm CAR-DVB-PDMS85 µm CAR-PDMS65 µm DVB-PDMS	78	71	86

a percentage of total metabolites obtained using all eight fibers.

b substitution with the 85 µm PA fiber results in 92, 92, and 97% coverage, respectively.
